# YAP promotes multi-drug resistance and inhibits autophagy-related cell death in hepatocellular carcinoma via the RAC1-ROS-mTOR pathway

**DOI:** 10.1186/s12935-019-0898-7

**Published:** 2019-07-12

**Authors:** Yuan Zhou, Yubo Wang, Wuhua Zhou, Tianchi Chen, Qinchuan Wu, Vikram Kumar Chutturghoon, Bingyi Lin, Lei Geng, Zhe Yang, Lin Zhou, Shusen Zheng

**Affiliations:** 1 Division of Hepatobiliary and Pancreatic Surgery, Department of Surgery, First Affiliated Hospital, School of Medicine, Zhejiang University, Hangzhou, China; 2 Key Laboratory of Combined Multi-organ Transplantation, Ministry of Public Health, Hangzhou, China; 3 Key Laboratory of Organ Transplantation, Hangzhou, Zhejiang Province China; 4 Key Laboratory of the Diagnosis and Treatment of Organ Transplantation, CAMS, Hangzhou, China; 5 Collaborative Innovation Center for Diagnosis Treatment of Infectious Diseases, Zhejiang University, Hangzhou, China; 6 Department of Hepatobiliary and Pancreatic Surgery, Taihe Hospital, Hubei University of Medicine, Hubei, China

**Keywords:** Hepatocellular carcinoma, Yes-associated protein (YAP), Multi-drug resistance, Autophagy-related cell death

## Abstract

**Background:**

Multi-drug resistance is the major cause of chemotherapy failure in hepatocellular carcinoma (HCC). YAP, a critical effector of the Hippo pathway, has been shown to contribute to the progression, metastasis and invasion of cancers. However, the potential role of YAP in mediating drug resistance remains obscure.

**Methods:**

RT-qPCR and western blot were used to assess YAP expression in HCC cell lines. CCK-8 assays, flow cytometry, a xenograft tumour model, immunochemistry and GFP-mRFP-LC3 fusion proteins were utilized to evaluate the effect of YAP on multi-drug resistance, intracellular ROS production and the autophagy of HCC cells in vitro and in vivo. Autophagy inhibitor and rescue experiments were carried out to elucidate the mechanism by which YAP promotes chemoresistance in HCC cells.

**Results:**

We found that BEL/FU, a typical HCC cell line with chemoresistance, exhibited overexpression of YAP. Moreover, the inhibition of YAP by shRNA or verteporfin conferred the sensitivity of BEL/FU cells to chemotherapeutic agents through autophagy-related cell death in vitro and in vivo. Mechanistically, YAP silencing significantly enhanced autophagic flux by increasing RAC1-driven ROS, which contributed to the inactivation of mTOR in HCC cells. In addition, the antagonist of autophagy reversed the enhanced effect of YAP silencing on cell death under treatment with chemotherapeutic agents.

**Conclusion:**

Our findings suggested that YAP upregulation endowed HCC cells with multi-drug resistance via the RAC1-ROS-mTOR pathway, resulting in the repression of autophagy-related cell death. The blockade of YAP may serve as a promising novel therapeutic strategy for overcoming chemoresistance in HCC.

**Electronic supplementary material:**

The online version of this article (10.1186/s12935-019-0898-7) contains supplementary material, which is available to authorized users.

## Background

Hepatocellular carcinoma (HCC) is still one of the most prevalent malignancies, with a high rate of occurrence and death worldwide [1, 2] despite tremendous development in diagnosis and therapy. Surgical resection and liver transplantation provide a curable opportunity for HCC, but unfortunately, they are unsuitable for a large number of patients with advanced-stage HCC [3, 4]. These patients with advanced-stage HCC can benefit to some extent from alternative treatments, including transarterial chemoembolization (TACE), molecularly targeted therapy and transitional chemotherapy [5, 6].

However, these palliative treatments are often limited by the occurrence of chemoresistance, especially multi-drug resistance (MDR), in HCC [7]. Cancer cells with MDR are characterized by resistance to one chemotherapy agent along with cross-resistance to other agents with different structures and mechanisms [8, 9]. A variety of mechanisms are involved in MDR, including the overexpression of the ABC transporter family [10], epithelial-mesenchymal transition (EMT) [11], cancer cell regulation [12], apoptosis induction, DNA damage and repair, and autophagy induction [13]. Overcoming MDR during chemotherapeutic interventions for cancers is a great challenge [13]. Thus, it is urgent to uncover the exact molecular mechanisms of MDR.

The Hippo signalling pathway is crucial for diverse cellular processes, including proliferation, organ development, stem cell biology, cell survival and tumourigenesis [14–16]. This pathway is implicated in transcriptional activity by the regulation of its core downstream effector, yes-associated protein (YAP), which can be directly phosphorylated and inactivated by large tumour suppressor 1/2 (LATS1/2), resulting in the retention of YAP in the cytoplasm [17]. The attenuation of the Hippo pathway could induce YAP interacting with DNA-binding transcriptional factors and TEA domain family members (TEADs) and, subsequently, its translocation to the nucleus and activation of gene expression [18, 19]. Previous evidence has shown that high expression of YAP is closely related to the poor clinical outcome of malignancies [20, 21]. In addition, YAP has been determined to modulate cancer stem cell-like behaviours and EMT, both of which are crucial for MDR [22, 23]. These findings imply that YAP is a critical contributor to the progression of cancers and has a potential role in the development of MDR.

Here, we demonstrated that the upregulation of YAP promoted MDR by repressing autophagy-related cell death via modulating the RAC1-reactive oxygen species (ROS)-mTOR pathway and that targeting YAP might be an alternative to improving the sensitivity of HCC to chemotherapy.

## Results

### YAP was highly expressed in the multi-drug-resistant cell line BEL/FU and promoted BEL-7402 resistance to chemotherapy

**Fig. 1:**
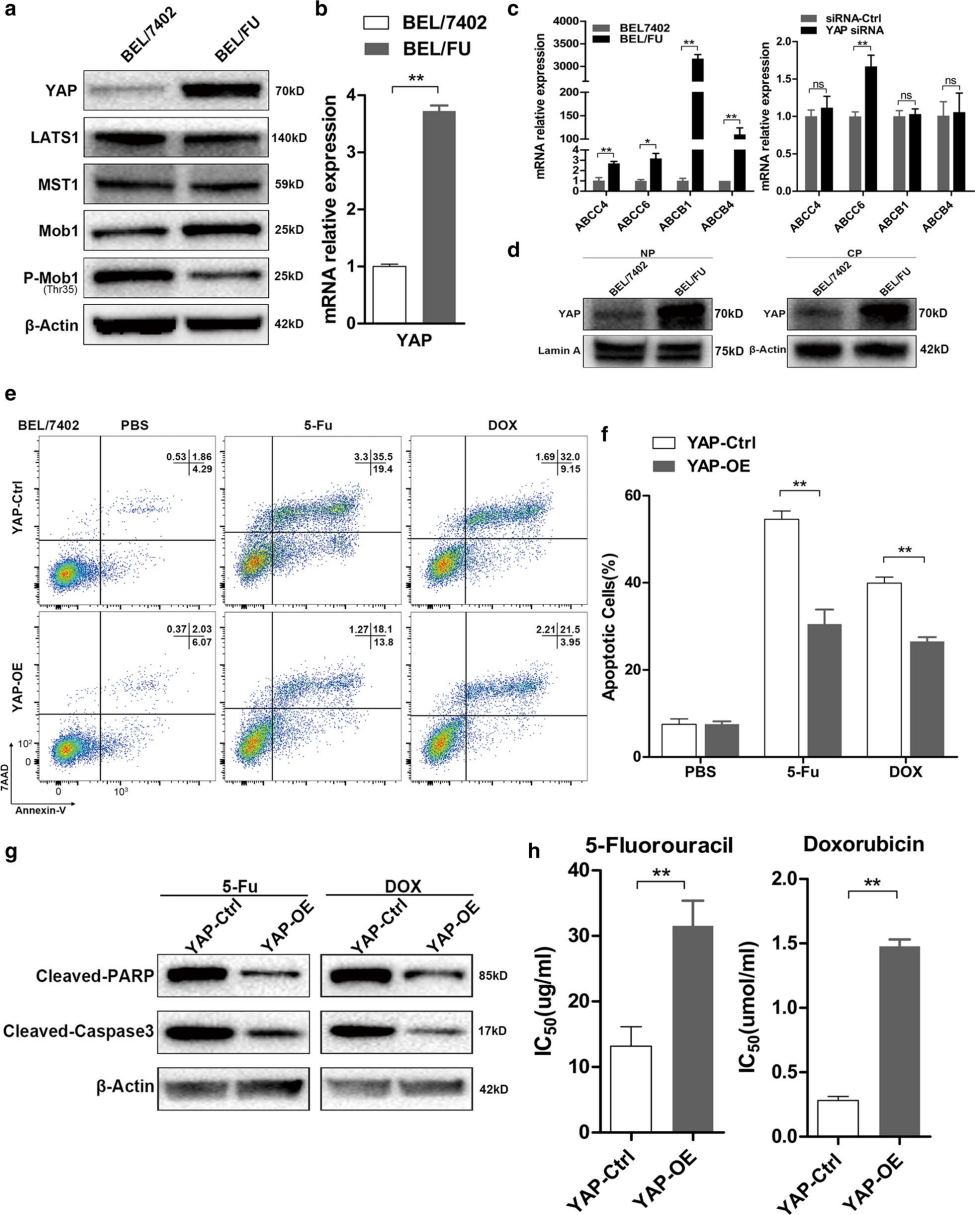
YAP is critical for the multi-drug resistance of HCC cell lines. **a** The protein expression of YAP and main components of the Hippo pathway in the BEL/FU and BEL-7402 cell lines were analysed by western blot. β-Actin was used as a loading control. **b** The mRNA level of YAP in the BEL/FU and BEL-7402 cell lines. **c** The mRNA level of ABCC4, ABCC6, ABCB1 and ABCB4 in BEL/FU and BEL/7402 cells (left panel). The mRNA level of ABCC4, ABCC6, ABCB1 and ABCB4 in BEL/FU cells with or without YAP knockdown (right panel). **d** Nuclear and cytoplasmic YAP expression in the BEL/FU and BEL-7402 cell lines. NP: nuclear protein, CP: cytoplasmic protein. β-Actin or lamin A were used as loading controls. **e**, **f** The percentage of apoptotic BEL-7402 cells with or without YAP overexpression after treatment with PBS, 5-Fu (20 µg/ml) or DOX (0.5 µmol/ml) for 48 h was analysed by flow cytometry. **g** The amount of the apoptosis markers, cleaved PARP and cleaved caspase-3, in BEL-7402 cells with or without YAP overexpression after treatment with 5-Fu (20 µg/ml) or DOX (0.5 µmol/ml) for 48 h was analysed by western blot analysis. **h** The IC_50_ values of 5-Fu and DOX in BEL-7402 cells with or without YAP overexpression (indicated as YAP-OE and YAP-Ctrl, respectively) were analysed by CCK-8 assay. Data are presented as the mean ± SD. *p < 0.05, **p < 0.01, ns, no significance

Our previous study confirmed that BEL/FU cells derived from the BEL-7402 cell line were simultaneously and stably resistant to 5-fluorouracil (5-Fu) and doxorubicin (DOX) [24]. We first compared the YAP and ATP-transports (ABCC4, ABCC6, ABCB1 and ABCB4) level in BEL/FU cells with that in the parent BEL-7402 cells by qRT-PCR and western blot assays. YAP was dramatically increased in BEL/FU cells at both the protein and mRNA levels (Fig. 1a, b). The mRNA expression of ABCC4, ABCC6, ABCB1 and ABCB4 was increased in BEL/FU cells significantly, whereas YAP knockdown did not decrease the RNA level of ATP-transports (Fig. 1c). In addition, multiple upstream inhibitors of YAP, including LATS1, Mob1 and p-Mob1, were attenuated in BEL/FU cells (Fig. 1a). Given that the biological function of YAP is ascribed to its nuclear-cytoplasmic translocation [17], it is necessary to detect the amount of YAP within the cytoplasm and nucleus. YAP accumulated within the nucleus of BEL/FU cells (Fig. 1d). These observations were further supported by reduced apoptosis, increased IC_50_ values, and decreased expression levels of cleaved caspase-3 and cleaved PARP in BEL-7402 cells with stable overexpression of YAP under treatment with 5-Fu or DOX (Fig. 1e–h).

### MDR was effectively overcome by YAP suppression in BEL/FU cells in vitro

**Fig. 2:**
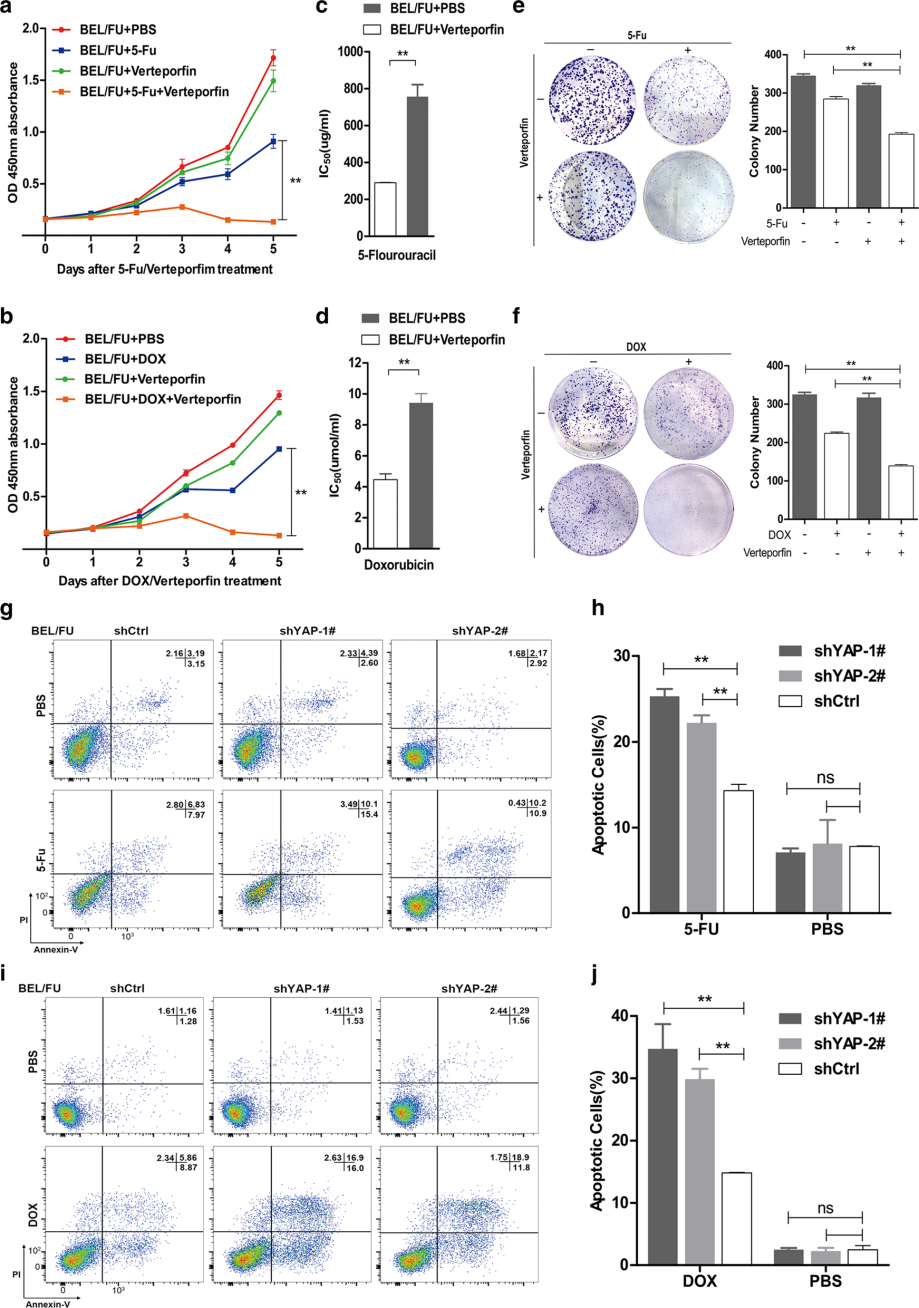
Inhibition of YAP overcame the multi-drug resistance of BEL/FU cells. **a**, **b** The assessment of the proliferation of BEL/FU cells after treatment with 5-Fu (0.3 mg/ml) or DOX (2 µmol/ml) with or without the YAP antagonist verteporfin (0.3 µg/ml) by CCK-8 assay. **c**, **d** The IC_50_ values of 5-Fu or DOX in BEL/FU cells in the presence of verteporfin or PBS. **e**, **f** The colony formation of BEL/FU cells was assessed under treatment with 5-Fu (0.3 mg/ml) or DOX (2 µmol/ml) with or without verteporfin (0.3 µg/ml). **g**–**j** Apoptosis was measured in BEL/FU cells with or without YAP knockdown after treatment with 5-Fu (0.3 mg/ml) or DOX (2 µmol/ml) for 48 h by flow cytometry. Data are presented as the mean ± SD. **p < 0.01

Next, we further investigated whether the MDR of BEL/FU cells could be alleviated by the inhibition of YAP. As expected, cell proliferation and colony formation were significantly suppressed by the effective YAP inhibitor verteporfin in BEL/FU cells treated with 5-Fu or DOX (Fig. 2a, b, e, f). Moreover, the sensitivity of BEL/FU cells to 5-Fu or DOX from YAP inhibition was substantiated by a decrease in IC_50_ values using the CCK-8 assay (Fig. 2c, d). In line with this finding, the percentage of apoptotic BEL/FU cells increased when YAP was stably knocked down by lenv-shRNA in the presence of 5-Fu or DOX (Fig. 2g–j; Additional file 1: Fig. S1A). However, YAP knockdown hardly altered the ratio of apoptotic BEL/FU cells without the treatment of 5-Fu or DOX. In addition, the amount of cleaved PARP and cleaved caspase-3 proteins was elevated by YAP knockdown in BEL/FU cells (Additional file 1: Fig. S1B).

### The YAP inhibitor verteporfin conferred the sensitivity of BEL/FU cells to chemotherapy in vivo

To validate the regulation of YAP on the chemoresistance of HCC in vivo, treatment with a combination of verteporfin and 5-Fu or DOX was conducted using a subcutaneous xenograft tumour model. The results demonstrated that tumour growth was moderately inhibited by treatment with 5-Fu, DOX or verteporfin alone but significantly suppressed by the combination of verteporfin and either 5-Fu or DOX (Fig. 4a). The group receiving a combination of verteporfin and 5-Fu or DOX showed tumour lesions of the smallest sizes and lowest weights (Fig. 4a–c). These data suggested that the YAP inhibitor verteporfin was a putative measure to effectively overcome the MDR of HCC cells.

In view of the DOX/5-Fu cytotoxicity dependent on the accumulation of intracellular ROS [25, 26], the chemoresistance mediated by ROS attenuation, the novel approach for overcoming MDR by increasing ROS or disrupting redox homeostasis [27], and the implication of Hippo-YAP signalling in the oxidative stress response [28, 29], we speculated that MDR was abrogated by the inhibition of YAP, likely due to the accumulation of intracellular ROS.

**Fig. 3:**
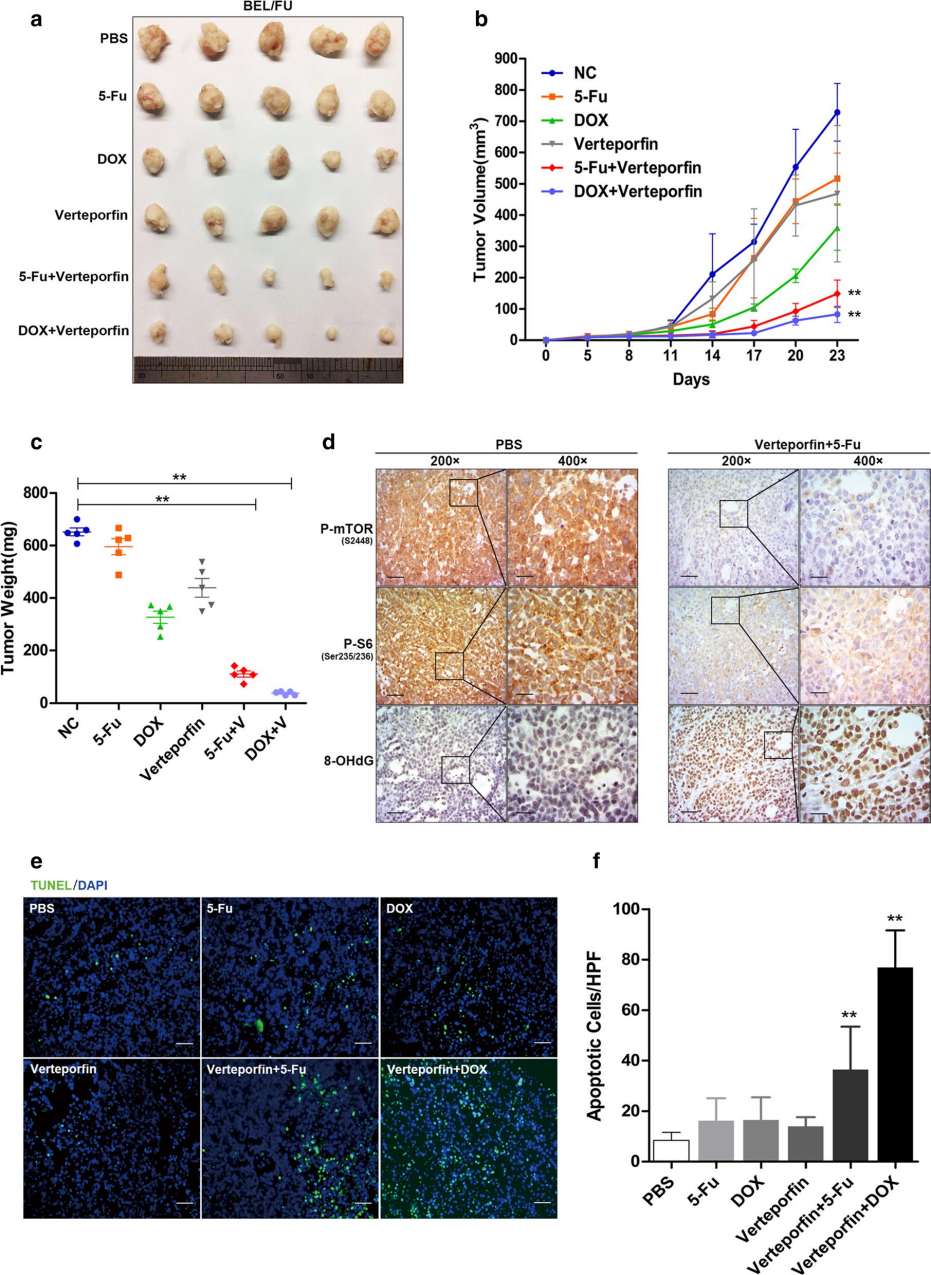
YAP conferred BEL/FU cells with chemoresistance in vivo. Balb/c nude mice with subcutaneous xenograft tumours were treated with PBS, 5-Fu (20 mg/kg), DOX (1 mg/kg), verteporfin (10 mg/kg) or a combination of verteporfin and 5-Fu or DOX intraperitoneally every 3 days. **a** Tumour appearance. **b** Tumour volumes. **c** Tumour weights. **d** The expression of p-mTOR, p-S6 and 8-OHdG was examined by IHC analysis of the tumour tissues (scale bar: 50 µm/25 µm). **e**, **f** Apoptosis was analysed in tumour tissues by terminal deoxynucleotidyl transferase dUTP nick end labelling (TUNEL) assay (scale bar: 50 µm). The average number of apoptotic cells from 10 random fields of magnification (200×). Data are presented as the mean ± SD. **p < 0.01

Of note, the most ROS production was detected in the xenograft tumour tissue of the group receiving the combination of verteporfin and 5-Fu or DOX, as indicated by strong staining of 8-hydroxy-2-deoxyguanosine (8-OHdG), an indicator of oxidative DNA damage (Fig. 3d; Additional file 1: Fig. S1F). Moreover, the apoptotic ratio in the tumour tissues was dramatically higher in the group of verteporfin combined with 5-Fu or DOX than in the other groups (Fig. 3e, f). The mammalian targeting of rapamycin (mTOR) signalling pathway is crucial for cell survival and can be significantly inhibited by increased ROS [30].

Here, immunohistochemistry (IHC) results showed the weakest staining of phosphorylated mTOR (p-mTOR) and phosphorylated S6 ribosomal protein (p-S6), two important activated proteins of mTOR signalling, in the tumour tissues of the group receiving the combination of verteporfin and 5-Fu or DOX (Fig. 3d; Additional file 1: Fig. S1D). This observation suggested that the mTOR signalling pathway was hampered by treatment with verteporfin together with 5-Fu or DOX. Collectively, our findings indicated that the co-treatment of verteporfin and 5-Fu or DOX promoted the sensitivity of HCC to chemotherapy by ROS accumulation and mTOR signalling cessation.

### YAP silencing promoted RAC1 upregulation, resulting in ROS production

**Fig. 4:**
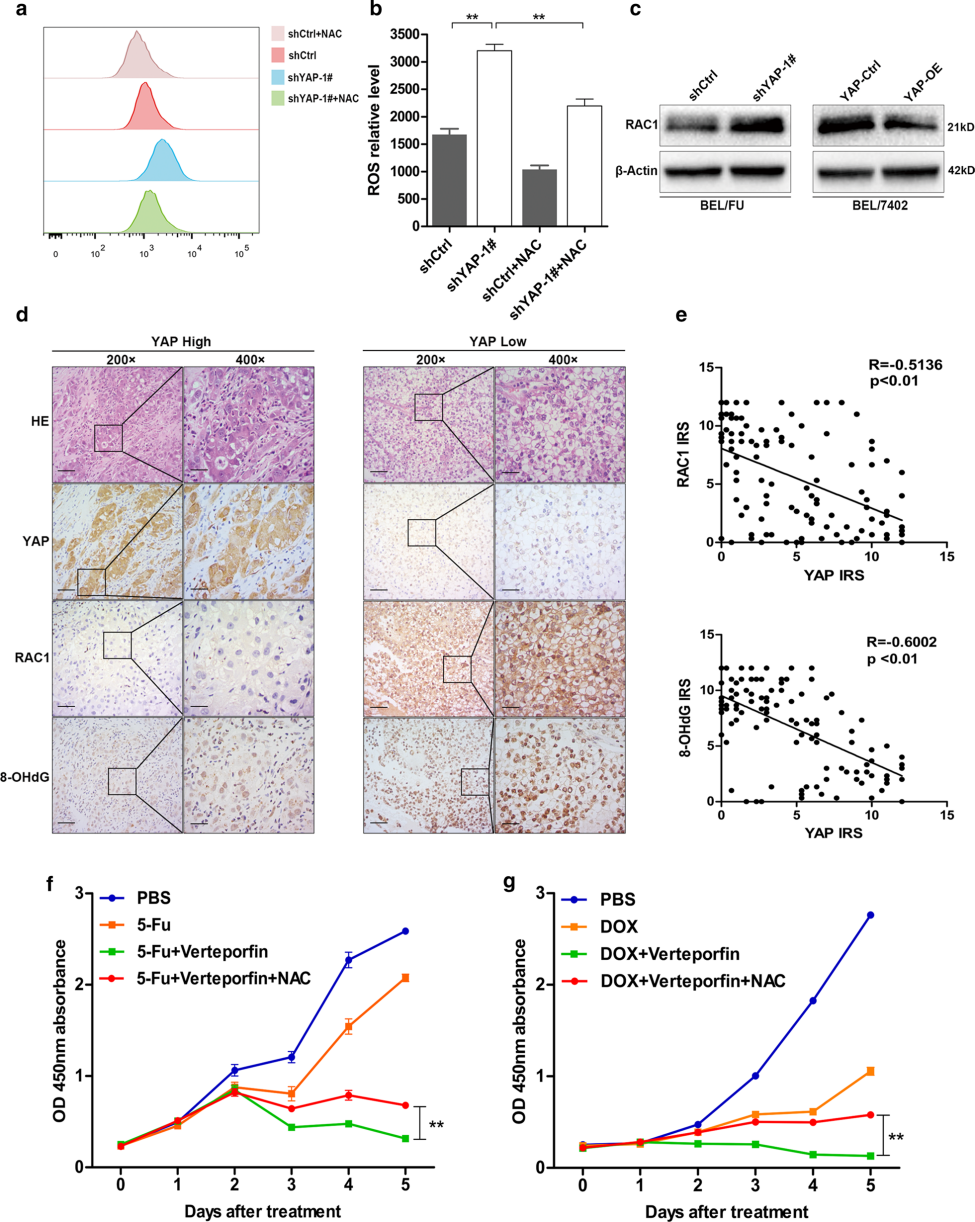
YAP downregulated RAC1, resulting in an attenuation of ROS production. **a**, **b** Intracellular ROS were examined in BEL/FU cells after treatment with 5-Fu (0.3 mg/ml) for 48 h through flow cytometry using CellROX™ Reagent. NAC (0.75 mM) was used for ROS clearance. **c** The protein level of RAC1 was examined in BEL/FU cells with YAP knockdown or overexpression by western blot. **d**, **e** The expression of YAP, RAC1 and 8-OHdG was detected in the HCC tissue microarray (n = 120) by IHC (scale bar: 50 µm/25 µm). The YAP level was negatively correlated with the expression of RAC1 and 8-OHdG based on the IRS. **f**, **g** NAC abolished the growth inhibition of BEL/FU cells induced by the combined treatment of verteporfin (0.3 µg/ml) and 5-Fu (0.3 mg/ml) or DOX (2 µmol/ml). Data are presented as the mean ± SD. **p < 0.01

To confirm the implication of YAP in suppressing ROS production, the amount of ROS in BEL/FU cells was detected with shYAP-1# treated by 5-Fu using flow cytometry in vitro. Under the treatment of 5-Fu, YAP knockdown significantly increased ROS production in the BEL/FU cells (Fig. 4a, b). ROS production is regulated by RAC1, a key molecule that modulates cell death, migration and proliferation [31]. Western blot analysis revealed that RAC1 expression was significantly upregulated by YAP silencing but attenuated by YAP overexpression. In addition, YAP expression was negatively correlated with RAC1 expression and ROS production by IHC assay based on a tissue microarray (Fig. 4c–e). Supporting this finding, the viability of BEL/FU cells could be effectively protected by the ROS inhibitor N-acetylcysteine (NAC) under combined treatment with verteporfin and 5-Fu or DOX (Fig. 4f, g). In summary, the upregulation of RAC1 mediated by YAP silencing was responsible for ROS production in BEL/FU cells.

### YAP inhibition robustly reversed chemoresistance with autophagy-related cell death

Since YAP overexpression could prevent ROS accumulation, which is involved in cell death related to the initiation of autophagy during chemotherapy [32, 33], the conversion of LC3B-I to LC3B-II (which is indicated by being in a cytosolic form to a membrane-bound lapidated form and is the hallmark of autophagy activation [30]) was assessed to ascertain the association of YAP with autophagy. Cell lines with a high (BEL/FU and SK-Hep1) or low (BEL-7402 and HCC-LM3) expression of YAP were used in the further studies (Additional file 1: Fig. S1D).

**Fig. 5:**
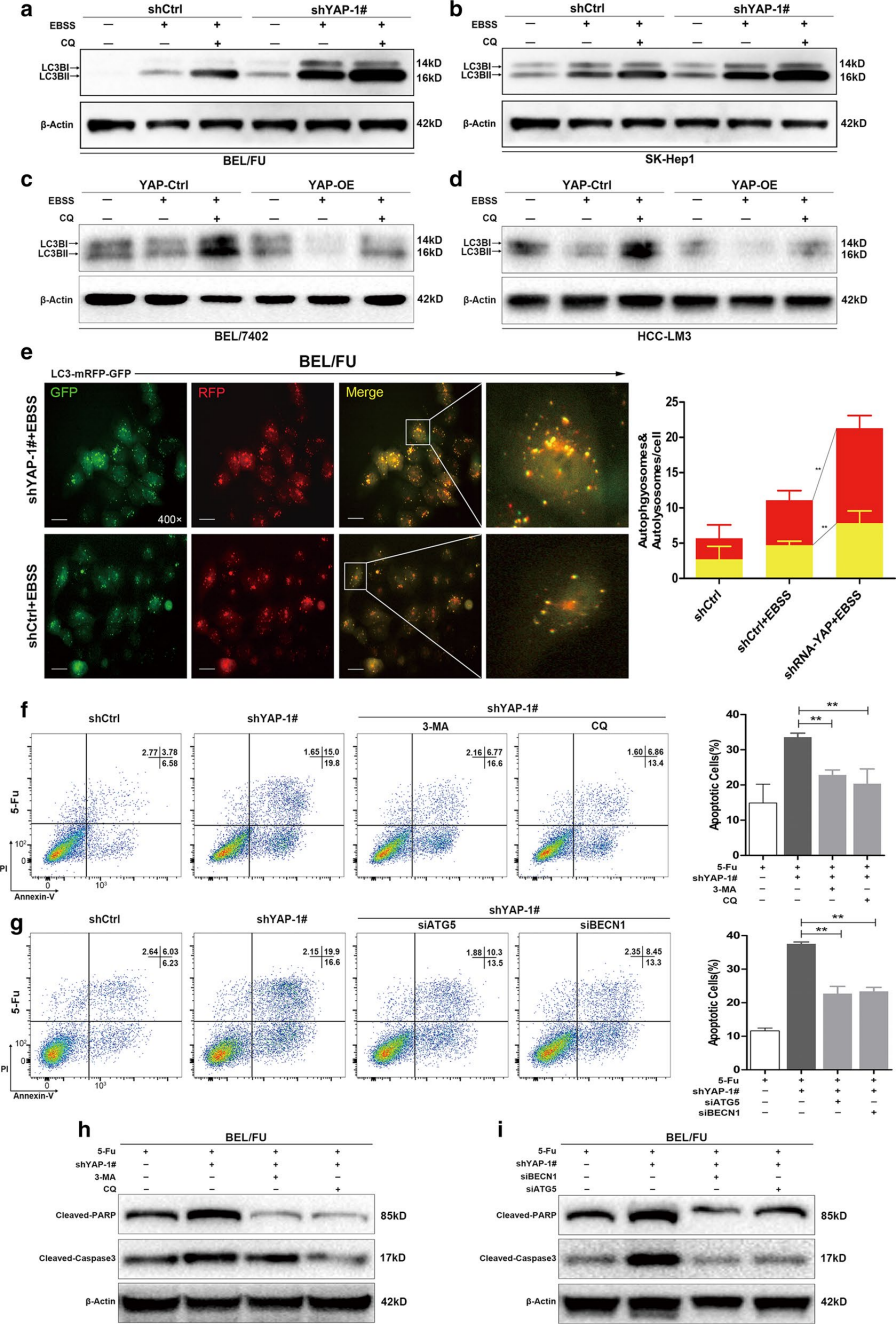
YAP knockdown facilitated autophagy and autophagy-related cell death in HCC cells. **a**–**d** The amount of the autophagy marker LC3B was measured in HCC cell lines (BEL/FU, SK-Hep1, BEL-7402 and HCC-LM3) with YAP knockdown or overexpression under conditions of complete medium or EBSS solution with or without CQ (100 µM) for 6 h. **e** The autophagosome (yellow puncta) and autolysosome (red puncta) were examined in BEL/FU cells with or without YAP knockdown after transfection of GFP-mRFP-LC3B fusion protein. Left panel, representative images. Right panel, quantification of autophagosomes and autolysosomes within a single cell (from 5 random fields) (scale bar: 25 µm). **f**–**i** BEL/FU cells with YAP knockdown were treated with the autophagy inhibitor 3-MA (5 mM) or CQ (100 µM) for 48 h or with the knockdown of ATG5 or BECN1 in the presence of 5-Fu (0.3 mg/ml). All cells were harvested for apoptosis analysis by flow cytometry (F, G) or detection of protein levels of cleaved PARP and cleaved caspase-3 by western blot **h**, **i**. β-Actin was used as a loading control. Data are presented as the mean ± SD. **p < 0.01

As shown in Fig. 5a–d and Additional file 2: Fig. S2A-D, the LC3B-II transition was elevated in BEL/FU and SK-Hep1 cells but reduced in BEL-7402 and HCC-LM3 cells under Earle’s Balanced Salt Solution (EBSS) starvation conditions in the presence or absence of chloroquine (CQ), an inhibitor of the fusion of autophagosomes with lysosomes. This observation was further confirmed by the tandem LC3B-RFP-GFP fluorescence microscopy assay, in which YAP shRNA remarkably increased the formation of autophagosomes and autolysosomes, implying that YAP knockdown enhanced autophagic flux (Fig. 5e).

We next evaluated the effect of YAP knockdown-induced autophagic flux on cell death in the presence of 5-Fu. The percentage of apoptotic cells and the protein levels of cleaved PARP and cleaved caspase-3 were significantly suppressed in BEL/FU cells with YAP knockdown after treatment with CQ or 3-methyladenine (3-MA), an inhibitor of the early stage of autophagy (Fig. 5f, h). In addition, the reduction in apoptotic cells, cleaved PARP and cleaved caspase-3 were observed in BEL/FU cells with YAP knockdown after a blockade of autophagy by siRNA against ATG5 and BECN1 (Fig. 5g, i; Additional file 1: Fig. S1C), both of which are critical autophagy-related genes [30]. These findings suggested that the reduction in autophagy-related cell death was responsible for YAP-mediated MDR in HCC.

### YAP-mediated ROS attenuation leads to an activation of the mTOR pathway, which is responsible for the inhibition of autophagy-related cell death

**Fig. 6:**
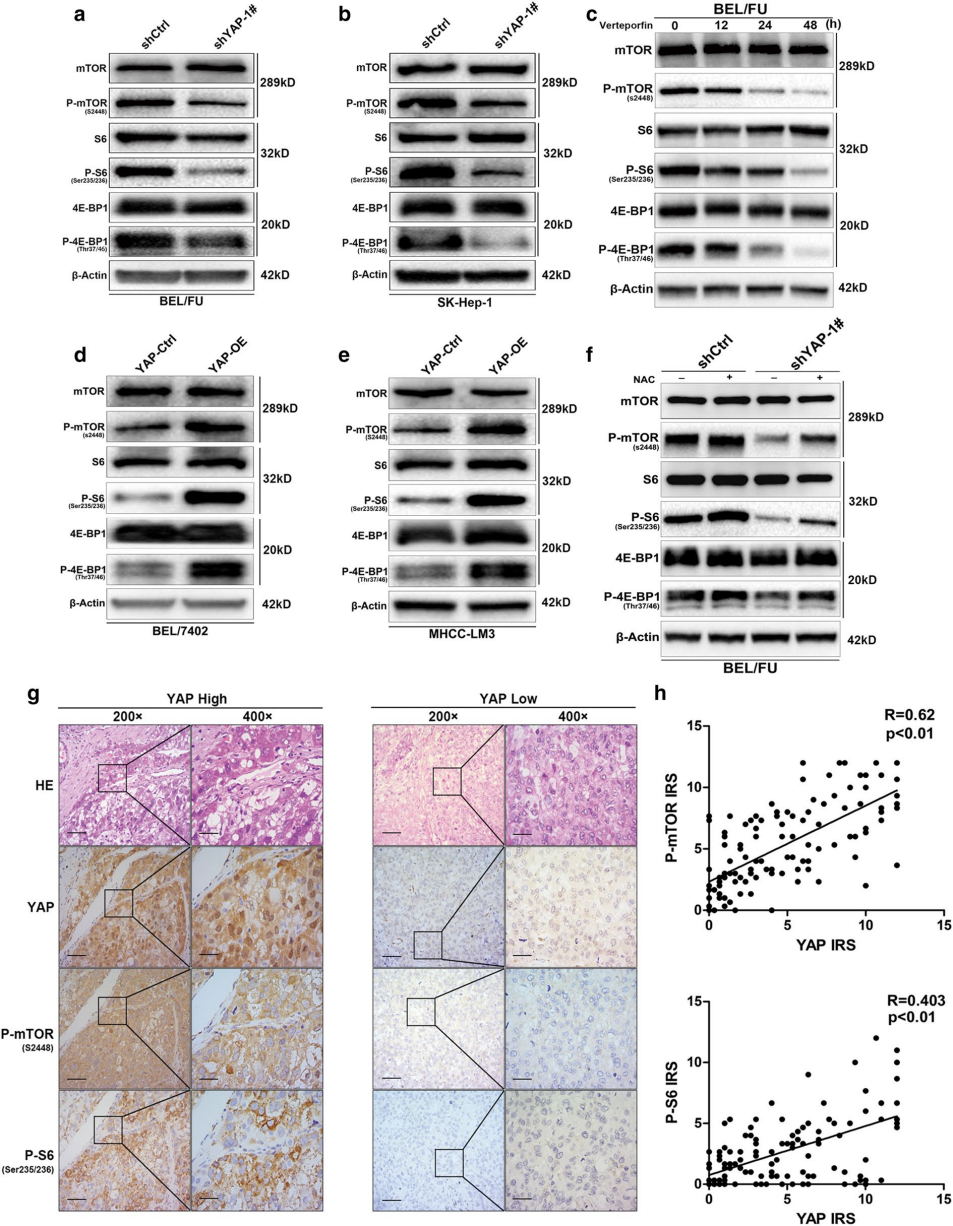
YAP regulated the activation of the mTOR pathway in HCC cells. **a**–**f** The protein levels of mTOR, p-mTOR, S6, p-S6, 4E-BP1 and p-4E-BP1 were analysed in HCC cell lines (BEL/FU, SK-Hep1, BEL-7402, and HCC-LM3) with YAP overexpression or inhibition by shRNA or the antagonist verteporfin. The inhibition of the mTOR pathway induced by YAP knockdown was partially reversed by NAC (0.75 mM) treatment. **g**, **h** The expression of YAP, p-mTOR and p-S6 was examined in the HCC tissue microarray (n = 120) by IHC (scale bar: 50 µm/25 µm). YAP levels were positively correlated with the expression of p-mTOR and p-S6 based on the IRS

mTOR is a canonical hub suppressor of autophagy and can be activated by the clearance of ROS [30]. mTOR is proposed to be regulated by YAP in the current study. First, the inhibition of mTOR by rapamycin significantly caused an increase in autophagic flux and cell death, evidenced by the increased transition to LC3B-II and increased protein levels of cleaved PARP and cleaved caspase-3 (Additional file 1: Fig. S1E, G). Second, YAP knockdown significantly reduced multiple activated proteins of mTOR signalling, including p-mTOR, p-S6 and phosphorylated translation repressor protein 4E-BP1 (p-4E-BP1), in the BEL/FU and SK-Hep-1 cell lines (Fig. 6a, b; Additional file 2: Fig. S2E, F). Treatment with the YAP antagonist verteporfin decreased the protein levels of p-mTOR, p-S6 and p-4E-BP1 in a time-dependent manner (Fig. 6c; Additional file 2: Fig. S2I). In contrast, these proteins were activated by YAP overexpression in the BEL-7402 and HCC-LM3 cell lines (Fig. 6d, e; Additional file 2: Fig. S2G, H). In addition, IHC analysis demonstrated that YAP expression had a positive correlation with the levels of p-mTOR and p-S6 (Fig. 6g, h). Third, the YAP knockdown-induced suppression of p-mTOR, p-S6 and p-4E-BP1 could be strikingly restored by the ROS scavenger NAC in BEL/FU cells (Fig. 6f; Additional file 2: Fig. S2J). Taken together, YAP knockdown was able to suppress the mTOR pathway through ROS production, leading to autophagy-related cell death.

## Discussion

Chemotherapy is widely accepted as one of the most common clinical treatments for advanced cancers. However, patients exposed to long-term chemotherapy are inclined to be unresponsive to chemo-drugs. MDR remains the major obstacle to successful and effective clinical treatment for cancer due to consequent metastasis and recurrence, both of which contribute to poor outcomes [24, 34]. Therefore, uncovering the potential mechanisms of MDR would provide a great opportunity to improve cancer chemotherapy. Here, we found that the upregulation of YAP through the inhibition of ROS-mediated autophagy-related cell death promotes MDR in HCC. Moreover, the blockade of YAP endowed HCC with sensitivity to chemotherapeutic agents.

More recent evidence has indicated that the accumulation of the YAP protein level is a substantial factor for micrometastases and resistance during chemotherapy in various types of cancer [34–36]. In line with these results, our data showed a high expression of YAP in BEL/FU cells, a well-known HCC cell line with resistance to chemotherapy. These observations were further supported by the reversal of MDR in BEL/FU cells after the administration of the YAP antagonist verteporfin in vitro and in vivo. Additionally, the exogenous overexpression of YAP reduced the percentage of apoptotic cells and elevated IC_50_ values in BEL-7402 cells under 5-Fu or DOX treatment. In addition, targeting YAP was also able to sensitize various types of cancer cells to chemotherapeutic agents [37–39], reflecting that YAP can be recognized as an antitumour target, especially for refractory malignancies, including HCC.

Moreover, YAP is robustly able to sustain the antioxidant potential, cell survival and chemoresistance of bladder cancer [40], relying on the vulnerability of cancer cells to ROS [41]. The suppression of antioxidants or restoration of intracellular ROS levels are increasingly being accepted as useful strategies against malignancies with chemoresistance. This viewpoint is supported by the application of treatment with anthracyclines and paclitaxel, which could promote anticancer responses to some extent by increasing intracellular ROS levels [42]. However, such chemotherapy is prone to failure with drug-resistant malignancies due to their intensive capability of scavenging intracellular ROS [43]. Therefore, targeting YAP might be an ideal option for overcoming drug resistance in cancers, including HCC. Indeed, YAP knockdown exhibited a strong ability to raise intracellular ROS production and resensitized BEL/FU cells to 5-Fu or DOX in the current study. This result was further substantiated by the negative relation of YAP expression with ROS levels in HCC tissues and the restored proliferation of BEL/FU cells with YAP silencing after treatment with the ROS scavenger NAC in the context of 5-Fu. The process of YAP-mediated ROS reduction and chemoresistance is dependent on RAC1 suppression, a critical regulator implicated in oxidation and ROS production [31]. In addition, this work further validated that the combination of the YAP inhibitor verteporfin and 5-Fu could lead to ROS production and the inhibition of tumour growth in vivo. Thus, the YAP-inhibited induction of ROS accumulation would be a promising mechanism to overcome chemoresistance or improve the treatment of refractory cancers.

Intracellular ROS accumulation could negatively regulate multiple survival signalling pathways and enhance the autophagic flux of cancer cells [44]. The mTOR protein, which plays a key role in suppressing autophagy and sustaining cell growth, is easily inactivated by exogenous H_2_O_2_ [42]. Considering these findings, mTOR and the autophagy process are thought to be involved in the YAP-mediated chemoresistance of BEL/FU cells. Noticeably, our data revealed that the activation of mTOR protein was markedly suppressed by YAP knockdown and subsequently restored by the ROS scavenger NAC. Furthermore, YAP silencing caused an increase in the LC3B-I/II transition (representing the formation of autophagosomes) and the number of autophagosomes, as indicated by yellow dots using the tandem LC3B-mRFP-GFP fluorescence assay. Although autophagy has been reported as a protective factor for the initiation, progression and chemoresistance of cancers, increasing evidence has revealed that elevated and prolonged autophagic flux facilitates cell death and serves as a novel mechanism of tumour suppression during antitumour therapy [45–47]. The blockade of autophagy has been determined to enhance the resistance of cancer cells to antitumour agents, including imatinib, gefitinib and erlotinib, by promoting cell death [48, 49]. In accordance, our study demonstrated that the apoptotic cell ratio of BEL/FU cells was significantly reduced by the autophagy inhibitors CQ and 3-MA or the siRNA against BECN1 or ATG5 in the context of YAP silencing and 5-Fu treatment. Moreover, the cell death of BEL/FU cells was facilitated by a rapamycin-induced activation of autophagy, which is in agreement with the findings of a previous study in which the combination of rapamycin and temozolomide could overcome temozolomide resistance in human gliomas [50].

Despite the “two faces” role of autophagy, consisting of pro- and antitumour activities in tumourigenesis and the development of chemoresistance [48, 49], numerous studies have attempted to improve the sensitivity of breast cancer to tamoxifen or overcome chemoresistance by combining therapy with the enhancement of autophagic flux [48]. Increasing evidence suggests that capecitabine and gemcitabine, two agents widely used for colon, breast and pancreatic cancers, are able to suppress tumour progression by trigging excessive autophagic flux and autophagy-related cell death [51]. Considering that autophagy is a context-dependent process that is regulated by various factors, including the microenvironment and cell type and status, more knowledge is needed to clarify the role of autophagy in chemoresistance and cell death as well as in the survival of patients and efficacy of a combination of autophagy antagonists and chemotherapeutic agents for malignancies in the future.

## Conclusion

**Fig. 7:**
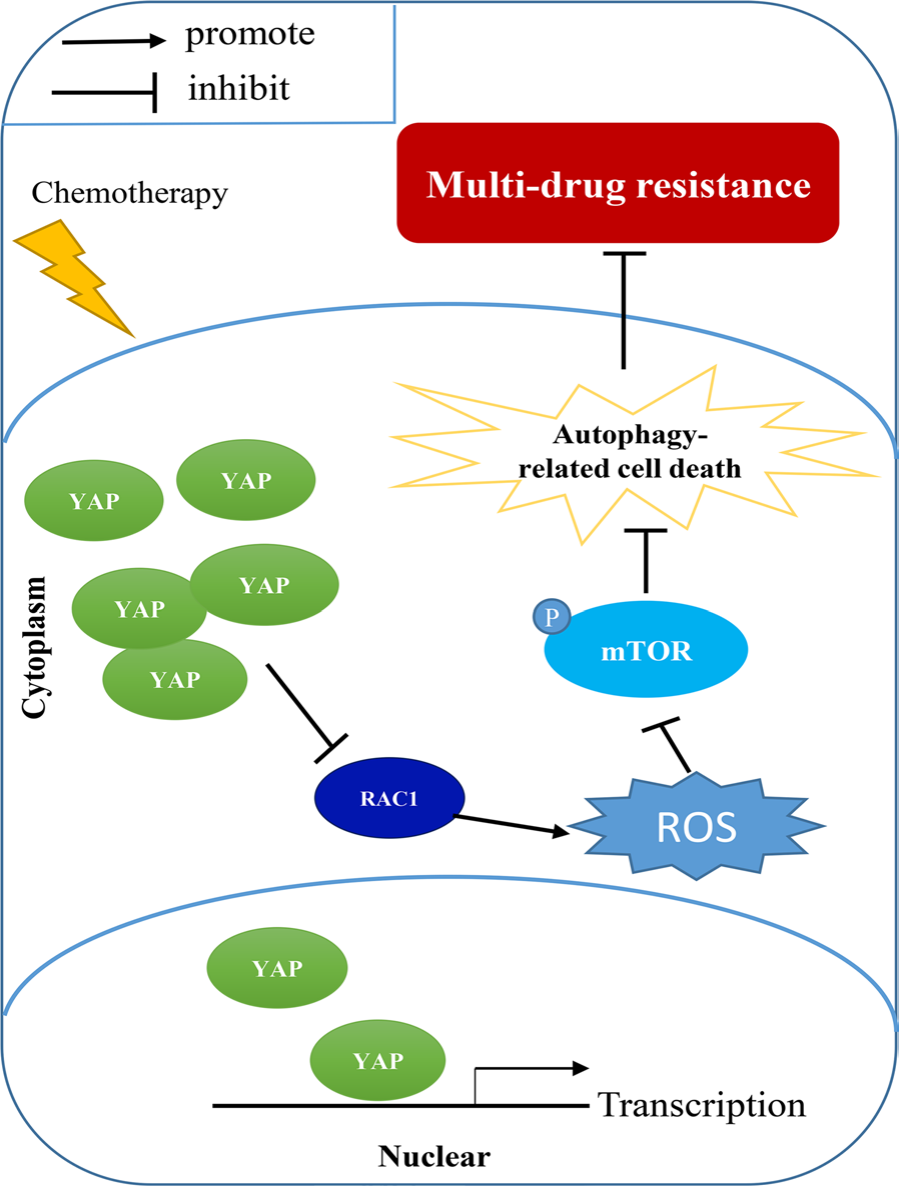
Schematic model representing the mechanism of YAP promotes multi-drug resistance in HCC. P: Phosphorylation

In summary, we believe that the dysregulation of intracellular ROS levels induced by chemotherapeutic agents was able to damage cancer cells through the activation of autophagy-related cell death; however, the upregulation of YAP in drug-resistant cells inhibits ROS production and maintains the activation of mTOR protein, thereby blocking the process of autophagy-related cell death and promoting chemoresistance (Fig. 7).

## Materials and methods

### Cell lines and reagents

The human HCC cell line BEL/FU and the parent cell line BEL-7402 were obtained from KeyGen Biotech Co., Ltd. (Nanjing, China). The BEL/FU cells and BEL-7402 cells were cultured in RPMI-1640 (Biological Industries, Israel) containing 10% foetal bovine serum (Biological Industries, Israel). The HCC-LM3 cell line was provided by the Cell Bank of the Shanghai Institutes of Biological Science, Chinese Academy of Sciences and cultured in DMEM high glucose medium (Biological Industries, Israel) with 10% foetal bovine serum (Biological Industries, Israel). SK-Hep-1 cells were provided by the China Center for Type Culture Collection and cultured in DMEM containing 10% foetal bovine serum at 5% CO_2_ and 37 °C. EBSS (Gibco) was used to provide starvation conditions. CQ (Selleck, USA) and 3-MA (Selleck, USA) were used to block autophagy. Verteporfin, 5-Fu, and DOX were purchased from MedChemExpress (MCE, USA).

### Colony formation assay

A colony formation assay was performed by seeding BEL/FU cells in 6-well plates. After treatment with 5-Fu, DOX and verteporfin for 48 h, the cells were cultured in fresh medium for 14 days. Then, the colonies were stained with crystal violet for counting.

### In vitro drug cytotoxic and cell proliferation assay

The drug cytotoxic assay was performed similar to a previous research method [24]. IC_50_ values were calculated by probit regression using SPSS software. To assess cell proliferation, BEL/FU cells (1 × 10^3^ cells/well) were placed into 96-well plates and measured at 0, 24, 48, 72, 96 and 120 h by CCK-8 (DOJINDO Laboratories, Japan) according to the manufacturer’s instructions.

### Clinical specimens

All HCC specimens contained in the tissue microarray were obtained from the First Affiliated Hospital, Zhejiang University School of Medicine, China. The tissues were fixed in formalin and embedded with paraffin.

### Immunohistochemistry

The sections of tissue microarrays and xenograft tumour tissues were deparaffinized in xylene. The primary antibodies against YAP (Abcam, USA), RAC1 (Proteintech, China), 8-OHdG (Abcam, USA), p-mTOR (Abcam, USA) and p-S6 (Abcam, USA) were used at suitable concentrations and incubated overnight at 4 °C. The intensity of staining was calculated according to the immunoreactive score system (IRS) [52].

### Western blot

Total protein was extracted from cells by using RIPA lysis buffer (Thermo, USA) with protease and phosphatase inhibitors (Thermo Scientific™, USA) on ice. The cell lysates from each sample were separated by 4–20% gradient sodium dodecyl sulphate (SDS)-polyacrylamide gel electrophoresis (GenScript, Nanjing, China) and then transferred onto 0.45 µm PVDF membranes (Millipore, USA). All membranes were blocked with Tris-buffered saline with 5% non-fat milk and 0.1% Tween-20 and then incubated with primary antibodies at 4 °C overnight. The secondary antibody was added and incubated for 1 h at room temperature at a concentration of 1:5000. The immunoblot bands were visualized using ECL kits (Proteintech, China). The protein amount was quantified by analysis of density of band using Image Lab software.

### Xenograft model assay

To determine the function of YAP in MDR in vivo, 4-week-old Balb/c nude mice were purchased from Shanghai X-B Animal Ltd., China. A total of 3 × 10^6^ BEL/FU cells were inoculated subcutaneously into the right flank of each mouse. After the appearance of a tumour mass, all mice were treated with 5-Fu (20 mg/kg, i.p., every 3 days) and/or verteporfin (10 mg/kg, i.p., every 3 days). Tumour size was monitored regularly, and tumour volume was calculated according to the formula (volume = length × width^2^)/2. All mice were sacrificed after 3 weeks of measurement. This xenograft assay was approved by the Ethics Committee for Laboratory Animals of the First Affiliated Hospital, Zhejiang University School of Medicine, Zhejiang, Hangzhou, China.

### Lentivirus transfection

To generate HCC cells with a stable YAP knockdown, lentiviral particles with YAP-specific shRNA (shYAP-1# and shYAP-2#, GeneCopeia, Guangzhou, China) lentiviral particles were transfected into SK-Hep-1 and BEL/FU cell lines. The sequences of shRNAs targeting YAP were 5′-GGAATTGAGAACAATGACGAC-3′ (shYAP-1#) and 5′-GGATACAGGTGATACTATCAA-3′ (shYAP-2#). For the overexpression of YAP, lentivirus-Flag-YAP-expressing vectors (Genechem Co., Ltd., Shanghai, China) were transfected into BEL-7402 and HCC-LM3 cells. All transfected cells were treated with puromycin (5 µg/ml).

### ROS detection

BEL/FU cells were cultured in RPMI-1640 with 5-Fu (0.3 mg/ml, 48 h) and then harvested and washed with phosphate-buffered saline (PBS). Afterwards, the cells were resuspended in PBS and incubated with 2 µl of CellROX Green Reagent (Thermo Scientific, USA) for 30 min. The amount of intracellular ROS, indicated as fluorescence intensity, was measured by flow cytometry.

### Statistical analysis

All experiments were independently conducted in triplicate. Statistical analysis was conducted using SPSS 17.0 software (Chicago, USA). Student’s *t* test was carried out to compare the differences between the two groups. The correlation among the expression of YAP, p-mTOR, p-S6, 8-OHdG and RAC1 was evaluated by calculating the Pearson’s correlation. P < 0.05 was identified as statistically significant, * indicates p < 0.05, and ** indicates p < 0.01.

## Additional files

**Additional file 1: Figure S1.** (A) The efficiency of YAP knockdown in BEL/FU cells. (B) The protein levels of cleaved PARP and cleaved caspase-3 were detected in BEL/FU cells with or without YAP knockdown after treatment with 5-Fu or DOX for 48 h by western blot. The protein amount of cleaved PARP and cleaved caspase-3 were measured and quantified by analysis of densitometry. (C) The efficiency of ATG5 and BECN1 knockdown in BEL/FU cells. (D) The mRNA expression of YAP in HCC cell lines and the protein expression of YAP in HCC-LM3 and SK-Hep1 cells. (E) The protein level of the autophagy marker LC3B was measured in BEL/FU cells with or without treatment with rapamycin (20 nM) for 6 h by western blot analysis. (F) The expression of p-mTOR, p-S6 and 8-OHdG was examined by IHC analysis of xenograft tumour tissues from Balb/c nude mice treated with verteporfin and DOX (scale bar: 50 µm/25 µm). (G) After treatment with 5-Fu and DOX, the amount of apoptosis markers, cleaved PARP and cleaved caspase-3, in BEL/FU cells with or without treatment with rapamycin for 48 h was measured by western blot. The protein amount of cleaved PARP and cleaved caspase-3 were measured and quantified by analysis of densitometry. Data are presented as the mean ± SD. *p < 0.05, **p < 0.01.

**Additional file 2: Figure S2.** (A-D) The protein amount of LC3B-II/LC3B-I was measured and quantified in HCC cell lines (BEL/FU, SK-Hep1, BEL-7402, and HCC-LM3) with YAP overexpression or knockdown under Earle’s Balanced Salt Solution (EBSS) starvation conditions in the presence or absence of chloroquine (CQ). (E–G) The protein amount of p-mTOR, p-S6 and p-4E-BP1 was measured and quantified in HCC cell lines (BEL/FU, SK-Hep1, BEL-7402, and HCC-LM3) with YAP overexpression or knockdown. (I, J) The protein amount of p-mTOR, p-S6 and p-4E-BP1 was measured and quantified in BEL/FU cells with verteporfin treatment and BEL/FU cells with or without YAP knockdown after treatment with NAC. CM: complete medium. Data are presented as the mean ± SD. *p < 0.05, **p < 0.01, ns, no significance.

## Data Availability

Not applicable.

## Abbreviations

HCC: hepatocellular carcinoma
MDR: multi-drug resistance
TACE: transarterial chemoembolization
EMT: epithelial-mesenchymal transition
5-Fu: 5-fluorouracil
DOX: doxorubicin
ROS: reactive oxygen species
NAC: *N*-acetylcysteine amide
3-MA: 3-methyladenine
CQ: chloroquine
IHC: immunohistochemistry
