# Supplementary figures and images for: YAP promotes multi-drug resistance and inhibits autophagy-related cell death in hepatocellular carcinoma via the RAC1-ROS-mTOR pathway
Source: Cancer Cell Int. 2019 Jul 12;19:179. doi: 10.1186/s12935-019-0898-7 (PMC6626386; doi:10.1186/s12935-019-0898-7)

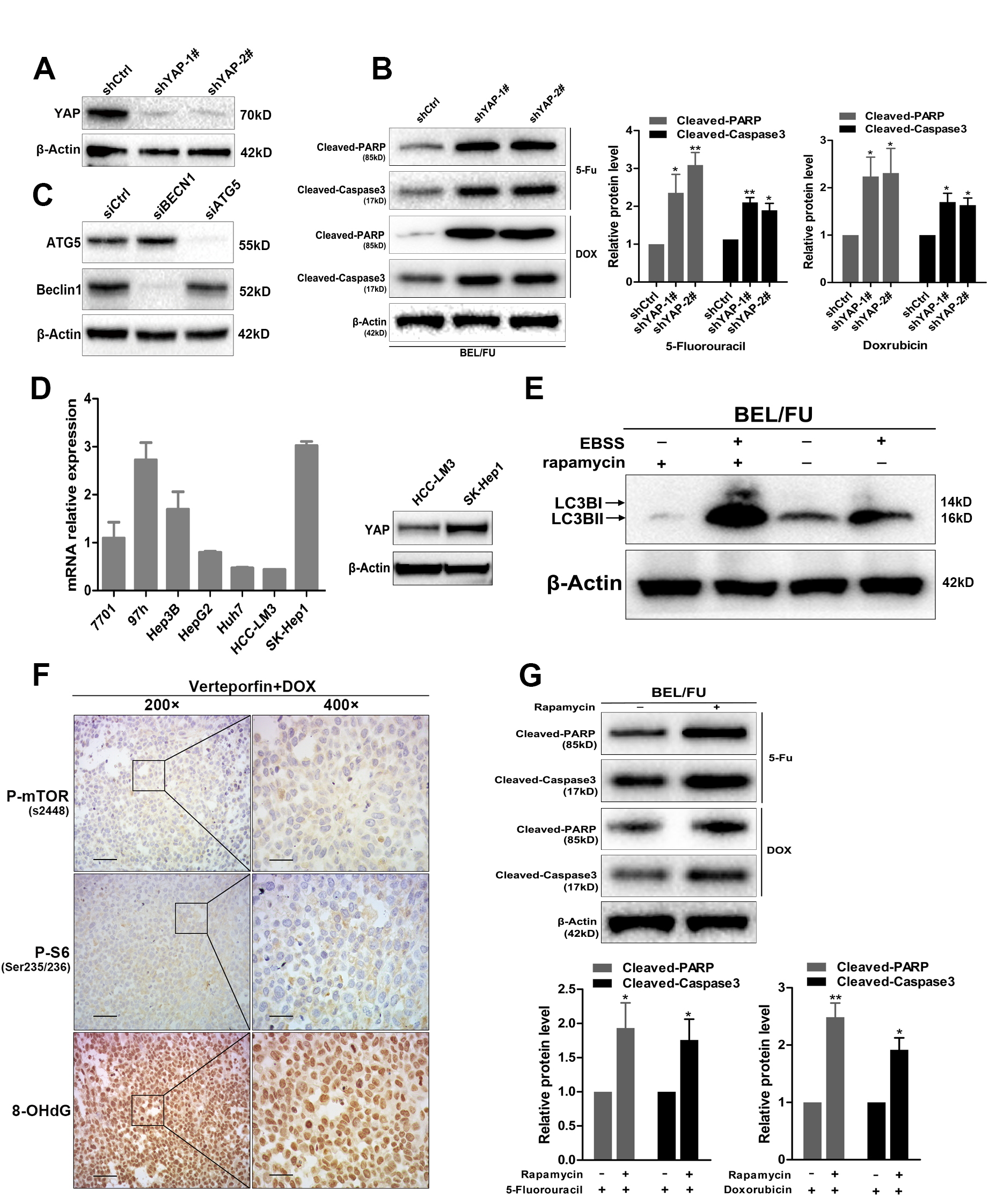

Supplement: Supplementary file 1 — Additional file 1: Figure S1. (A) The efficiency of YAP knockdown in BEL/FU cells. (B) The protein levels of cleaved PARP and cleaved caspase-3 were detected in BEL/FU cells with or without YAP knockdown after treatment with 5-Fu or DOX for 48 h by western blot. The protein amount of cleaved PARP and cleaved caspase-3 were measured and quantified by analysis of densitometry. (C) The efficiency of ATG5 and BECN1 knockdown in BEL/FU cells. (D) The mRNA expression of YAP in HCC cell lines and the protein expression of YAP in HCC-LM3 and SK-Hep1 cells. (E) The protein level of the autophagy marker LC3B was measured in BEL/FU cells with or without treatment with rapamycin (20 nM) for 6 h by western blot analysis. (F) The expression of p-mTOR, p-S6 and 8-OHdG was examined by IHC analysis of xenograft tumour tissues from Balb/c nude mice treated with verteporfin and DOX (scale bar: 50 µm/25 µm). (G) After treatment with 5-Fu and DOX, the amount of apoptosis markers, cleaved PARP and cleaved caspase-3, in BEL/FU cells with or without treatment with rapamycin for 48 h was measured by western blot. The protein amount of cleaved PARP and cleaved caspase-3 were measured and quantified by analysis of densitometry. Data are presented as the mean ± SD. *p < 0.05, **p < 0.01. [file 12935_2019_898_MOESM1_ESM.tif]

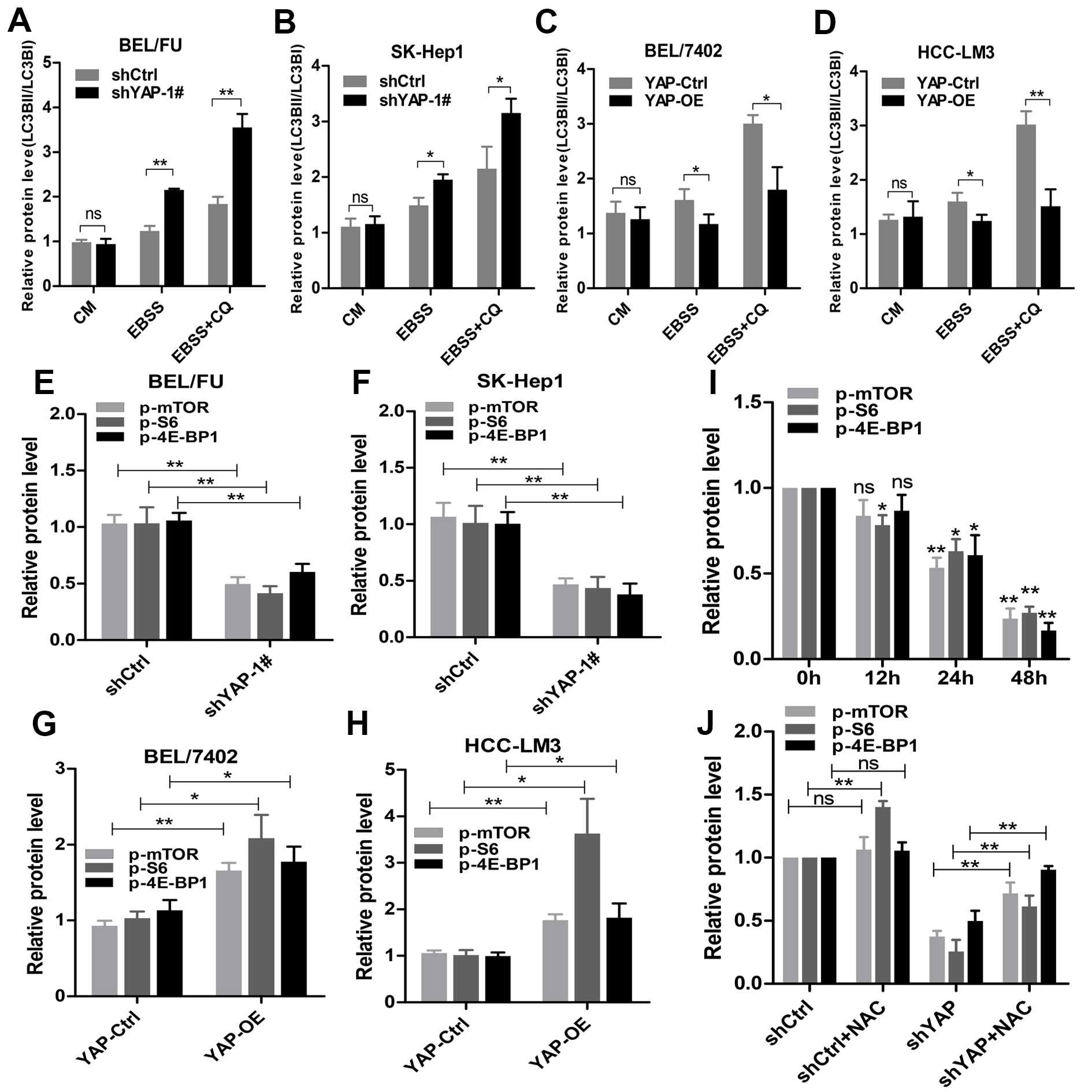

Supplement: Supplementary file 2 — Additional file 2: Figure S2. (A-D) The protein amount of LC3B-II/LC3B-I was measured and quantified in HCC cell lines (BEL/FU, SK-Hep1, BEL-7402, and HCC-LM3) with YAP overexpression or knockdown under Earle’s Balanced Salt Solution (EBSS) starvation conditions in the presence or absence of chloroquine (CQ). (E–G) The protein amount of p-mTOR, p-S6 and p-4E-BP1 was measured and quantified in HCC cell lines (BEL/FU, SK-Hep1, BEL-7402, and HCC-LM3) with YAP overexpression or knockdown. (I, J) The protein amount of p-mTOR, p-S6 and p-4E-BP1 was measured and quantified in BEL/FU cells with verteporfin treatment and BEL/FU cells with or without YAP knockdown after treatment with NAC. CM: complete medium. Data are presented as the mean ± SD. *p < 0.05, **p < 0.01, ns, no significance. [file 12935_2019_898_MOESM2_ESM.tif]
